# Supplementary material for: Structural Insight into La0.5Ca0.5Mn0.5Co0.5O3 Decomposition in the Methane Combustion Process
Source: Nanomaterials (Basel). 2021 Sep 2;11(9):2283. doi: 10.3390/nano11092283 (PMC8468899; doi:10.3390/nano11092283)
Supplement: Supplementary file 1 [file nanomaterials-11-02283-s001.zip › nanomaterials-1355696-Supplementary Materials.pdf]

Supplementary Materials

Structural Insight into $La_{0.5}Ca_{0.5}Mn_{0.5}Co_{0.5}O_3$ Decomposition in the Methane Combustion Process

Olga Nikolaeva, Aleksandr Kapishnikov, Evgeny Gerasimov*

Boreskov Institute of Catalysis SB RAS, Novosibirsk, 630090, Russia;
ribka-99@mail.ru (O.N.), avl97@mail.ru (A.K.)
* Correspondence: gerasimov@catalysis.ru (E.G.)

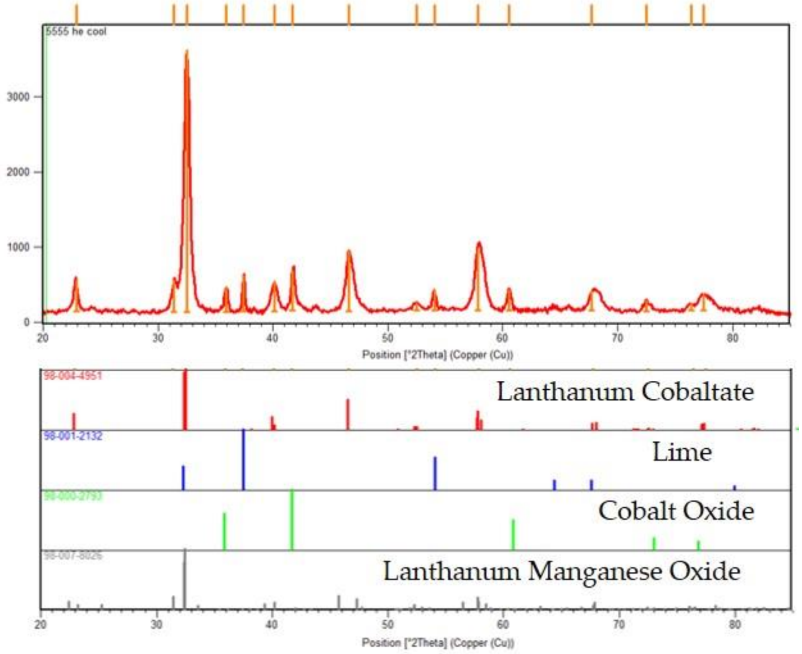

Figure S1. XRD pattern of La0.5Ca0.5Mn0.5Co0.5O3 sample after TGA in He atmosphere.

Pattern List

Visible	Ref.Code	Score	Compound Name
red	98-004-4951	76	Lanthanum Cobaltate
blue	98-007-8694	52	Cobalt Oxide
green	98-001-2132	42	Lime
gray	98-007-8026	39	Lanthanum Manganes

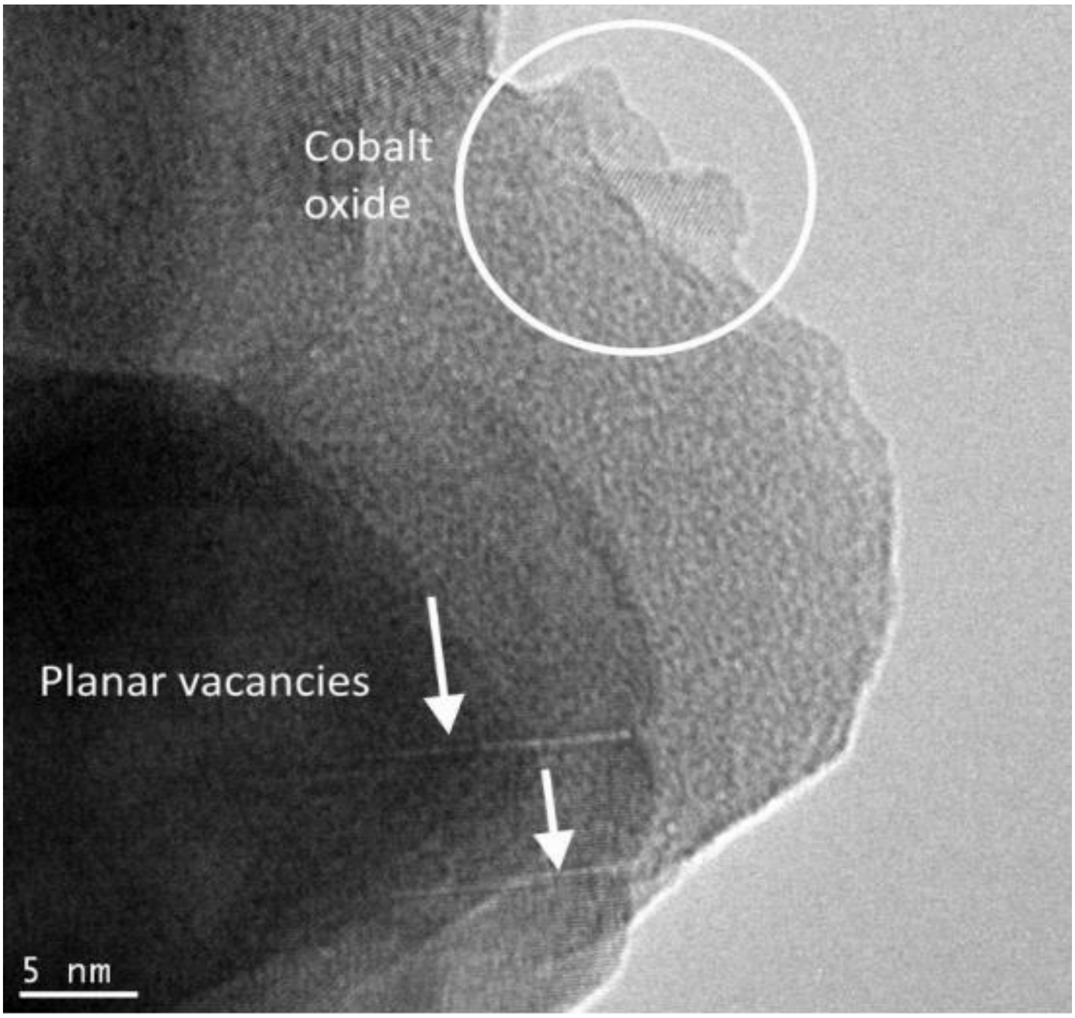

Figure S2. HRTEM image of the sample after the reaction. White arrows show planar vacancies. Co_3O_4 particles are shown by white circle.

The chemical equations of decomposition process in CH_4 and He are shown below:

$$La_{0.5}Ca_{0.5}Mn_{0.5}Co_{0.5}O_{3-\delta} + xCH_4 + (5x+y)O_2 = La_{0.5}Ca_{(0.5-x)}Mn_{0.5}Co_{(0.5-y)}O_{3-\delta} + xCaCO_3 + yCoO + (2x)H_2O, \quad (S1)$$

$$La_{0.5}Ca_{0.5}Mn_{0.5}Co_{0.5}O_{3-\delta} = La_{0.5}Ca_{(0.5-x)}Mn_{0.5}Co_{(0.5-y)}O_{3-\delta-x-y} + xCaO + yCoO \quad (S2)$$

The quasichemical equations of vacancies formation are shown below:

$$O_O^X + 2Co_{Co}^X \leftrightarrow V_O^{**} + 2Co_{Co}' + 1/2O_2 \quad (S3)$$

$$O_O^X + 2Mn_{Mn}^X \leftrightarrow V_O^{**} + 2Mn_{Mn}' + 1/2O_2 \quad (S4)$$
